# Investigation of volatile organic biomarkers derived from *Plasmodium falciparum in vitro*

**DOI:** 10.1186/1475-2875-11-314

**Published:** 2012-09-07

**Authors:** Rina PM Wong, Gavin R Flematti, Timothy ME Davis

**Affiliations:** 1 University of Western Australia, School of Medicine and Pharmacology, Fremantle Hospital, PO Box 480, Fremantle, WA, 6959, Australia; 2 University of Western Australia, School of Chemistry and Biochemistry, Nedlands, WA, Australia

**Keywords:** Malaria, *Plasmodium falciparum*, Volatile organic compounds, Solid phase micro-extraction, Organic biomarkers

## Abstract

**Background:**

There remains a need for techniques that improve the sensitive detection of viable *Plasmodium falciparum* as part of diagnosis and therapeutic monitoring in clinical studies and usual-care management of malaria infections. A non-invasive breath test based on *P. falciparum-*associated specific volatile organic compounds (VOCs) could fill this gap and provide insights into parasite metabolism and pathogenicity. The aim of this study was to determine whether VOCs are present in the headspace above *in vitro P. falciparum* cultures.

**Methods:**

A novel, custom-designed apparatus was developed to enable efficient headspace sampling of infected and non-infected cultures. Conditions were optimized to support cultures of high parasitaemia (>20%) to improve the potential detection of parasite-specific VOCs. A number of techniques for VOC analysis were investigated including solid phase micro-extraction using two different polarity fibres, and purge and trap/thermal desorption, each coupled to gas chromatography–mass spectrometry. Each experiment and analysis method was performed at least on two occasions. VOCs were identified by comparing their mass spectra against commercial mass spectral libraries.

**Results:**

No unique malarial-specific VOCs could be detected relative to those in the control red blood cell cultures. This could reflect sequestration of VOCs into cell membranes and/or culture media but solvent extractions of supernatants and cell lysates using hexane, dichloromethane and ethyl acetate also showed no obvious difference compared to control non-parasitized cultures.

**Conclusions:**

Future *in vivo* studies analysing the breath of patients with severe malaria who are harbouring a parasite biomass that is significantly greater than achievable *in vitro* may yet reveal specific clinically-useful volatile chemical biomarkers.

## Background

The detection of viable parasite forms is an essential requirement for malaria diagnosis and subsequent monitoring of the response to anti-malarial therapy. For diagnosis, microscopic examination of a peripheral blood smear remains the investigation of choice in a wide variety of clinical situations. However, the sensitivity of microscopy is limited even when expert microscopists view high quality slides. In addition, the diagnosis may be missed in cases of severe falciparum malaria in which the majority of parasites are sequestered within the microvasculature of major organs [1,2] or in the placenta in infected expectant mothers [3,4]. Antigen detection kits can be used where reliable microscopy is unavailable but their accuracy for placental malaria remains in question [5]. PCR increases diagnostic sensitivity but its timely availability is limited largely to specialized laboratories in developed countries. In addition, the sensitivity of PCR (down to 1 parasite/μL) means that even a child weighing only 15 kg and with a circulating blood volume of approximately 1 litre who is PCR-negative may still harbour up to a million malaria parasites. The monitoring of the response to antimalarial therapy in individual patients depends on the availability of serial blood smears complemented by PCR where available. Antigen detection methods cannot be used because of the persistence of antigen after parasite clearance, while PCR does not differentiate between DNA from viable and non-viable parasites [5].

There is a need for the development of alternative diagnostic tests that detect viable parasites before and after treatment with greater specificity and sensitivity than currently available methods. The human breath contains a large number of volatile organic compounds (VOCs) derived from the blood by passive diffusion in the lungs [6]. VOCs in the breath are directly related to concentrations in blood and other tissues as they flow from compartments with higher vapour pressure to those with lower pressure [6]. Breath tests have been used to assist in the early diagnosis of conditions such as heart disease, rheumatoid arthritis and lung cancer [7-9] as they detect increased VOCs released as a result of disease-specific cellular injury. More recently, exogenous VOCs produced by microorganisms such as *Mycobacterium tuberculosis* have been found in the breath of infected patients [10]. *Plasmodium* species may, in the same way, produce a characteristic VOCs ‘fingerprint’ that can facilitate diagnosis and therapeutic monitoring.

In the case of *Plasmodium falciparum* and perhaps *Plasmodium vivax *[11], the cytoadherence of mature parasite forms in the pulmonary microvasculature may facilitate detection of *Plasmodium*-specific VOCs in breath samples. The cause of altered consciousness in severe malaria remains unknown. VOCs are used as general anaesthetics in clinical practice [12] and it is possible that coma complicating malaria may result from elaboration of VOCs by malaria parasites in the cerebral microcirculation that have anaesthetic properties. In any case, malaria may, through indirect pathogenic tissue effects such as oxidative stress, alter the VOCs content of human breath in ways that are characteristic of the infection.

A number of extraction techniques are used for the capture and analysis of VOCs from human breath and the microbial culture atmosphere [13-17]. Sampling and sample preparation usually involve pre-concentrating the analytes of interest by purge and trap, headspace, liquid-liquid or solid phase extractions. These conventional techniques consist of multiple labour-intensive procedures and/or require organic solvents. Solid phase micro-extraction (SPME) is an adsorption/desorption technique that circumvents most of the drawbacks to sample preparation [17]. SPME can be used to concentrate volatile and non-volatile compounds in liquid samples or headspace without the use of solvents. The target compounds are subsequently separated and quantified by gas chromatography–mass spectrometry (GC-MS) with sensitivities down to parts per trillion levels [17].

The present study outlines the design and optimization of a *P. falciparum* culture-sampling system suitable for VOCs headspace capture and analysis. The VOCs emitted by *P. falciparum in vitro* as detected using GC-MS have been compared with those from control red blood cell cultures.

## Methods

### Parasites

**Figure 1:**
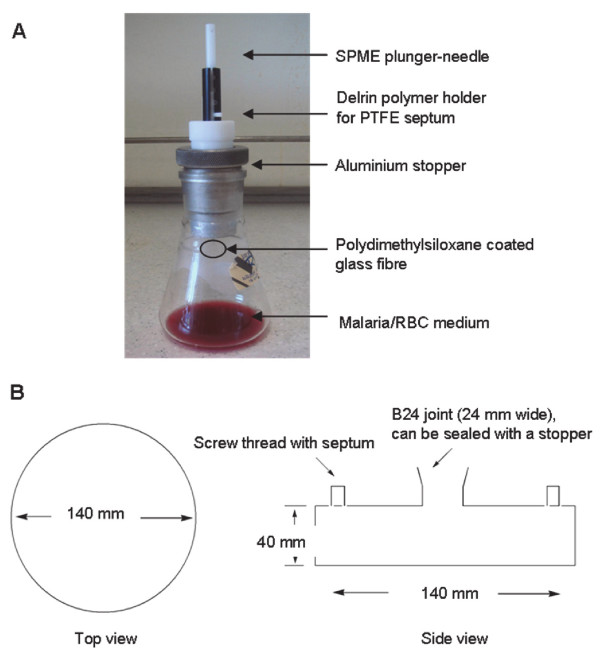
**Prototype 1 culture-capture apparatus with SPME is shown in panel A.** The prototype 1 design consisted of a 250 mL conical flask modified with a B-40 joint that served as a receptacle for an aluminium stopper. A delrin polymer SPME holder was screwed into the aluminium stopper and a syringe piercable polytetrafluoroethylene (PTFE) septum was placed between the mating surfaces to seal the sample before analysis. Design and dimensions of culture container (prototype 2) for headspace capture are shown in panel **B**. The wide-base (154 cm^2^) and shallow design maximizes culture efficiency and total parasite yield. The B-24 joint allows for easy access during RPMI addition and parasite sampling. Two side inlets facilitate the ease of low-oxygen gas-replacement and act as an access point for ‘purge and trap’ procedures and SPME of the headspace atmosphere.

The laboratory-adapted *P. falciparum* strains 3D7 (chloroquine-sensitive) and W2mef (chloroquine-resistant) were maintained in RPMI 1640 HEPES (Sigma Aldrich, St Louis, MO) supplemented with 92.6 mg/L L-glutamine (Sigma Aldrich) 500 μg/L gentamicin, 50 mg/L hypoxanthine (Sigma Aldrich) and 10% v/v pooled human plasma as previously described [18,19]. Once the parasitaemia was >5%, synchronous cultures at the trophozoite stage were transferred into custom-designed containers (Figure 1) at 1% haematocrit and purged with a mixture of 1% O_2_ and 5% CO_2_ in nitrogen at 5 psi for 4 sec and 30 sec for prototypes 1 and 2, respectively. Subsequent optimization used 5% O_2_ and 5% CO_2_ in nitrogen at 15 psi for 40 sec in the prototype 2 culture-sampling apparatus. The volume of media required to sustain high parasitaemia was calculated using the formula: volume of media (mL)/24 hr = 0.005 x (μL RBC pellet) x (% parasitaemia) [20]. This equation takes into account the nutrient requirements for non-parasitized as well as parasitized RBC. A control was set up with non-infected RBC using similar conditions and incubated for 24 hr at 37°C. 

### Solid phase micro-extraction

Volatile and semi-volatile compounds within the headspace of non-parasitized control and malaria cultures were pre-concentrated onto a SPME fibre coated with either polydimethylsiloxane (PDMS, 100 μM, #504823, SUPELCO, Bellefonte, PA, USA) or 50/30 μM Divinylbenzene/Carboxen/PDMS (triple fibre, #57328-U, SUPELCO, Bellefonte, PA, USA). The fibres were conditioned initially according to the manufacturer’s instructions (PDMS phase 250°C for 0.5 hr and triple fibre phase 270°C for 1 hr). Before each analysis, the fibre was activated in the injector port of the gas chromatograph (GC) at 250°C for 5 min and repeated after each sampling. The SPME fibre was introduced into the headspace of the container by gently pushing the protective needle through the septum that sealed the sample container. The plunger was lowered to expose the adsorbent fibre to the gaseous phase for one hour at 35°C. During this time, equilibrium between the atmosphere and the fibre was achieved, and the volatile and semi-volatile organic compounds were adsorbed onto the coating of the fibre. After sampling was completed, the fibre was retracted and the SPME fibre was manually loaded and injected into the GC injector port where VOCs were desorbed for 5 min in splitless mode at 250°C.

### Solvent extraction

Supernatants from 3D7 and W2mef cultures at high parasitaemia (5% to 13.2%) were pooled (100 mL and 150 mL, respectively). Cell pellets of each strain were lysed by sonication (Microson™ ultrasonic cell disruptor, Misonix Inc, NY) for 30 sec and diluted with distilled water prior to extraction. The aqueous supernatant was transferred into a separation funnel and partitioned 3 times with ⅓ volume of an organic solvent (hexane, dichloromethane or ethyl acetate). The procedure involved swirl-mixing with occasional depressurization via the outlet valve of the funnel. After the separation of aqueous and organic phases, the organic phase was back extracted against distilled water and collected into a conical flask followed by drying over anhydrous magnesium sulphate. After filtration (Whatman™ No. 1 filter paper), the solvent was evaporated under reduced pressure by means of a rotary evaporator (Rotavapor-R, Buchi Labortechnik, Flawil, Switzerland). For analysis by GC-MS, concentrated extracts were evaporated to dryness under a stream of nitrogen gas and resuspended in 100 μL of the extraction solvent.

### Purge and trap/ thermal desorption

For purge and trap extraction, non-parasitized control and *P. falciparum* infected RBC were cultured in prototype 2 containers (Figure 1). Air was drawn through the side inlets containing a loosely fitted cap and through the flask over the surface of the samples and the VOCs were trapped using a Tenax™ trap (200 mg Tenax TA 60/80, SUPELCO, Bellefonte, PA, USA). The headspace was collected for 1 hr at an airflow rate of 1.5 L/min using a portable air sampling pump (224-PCXR8, SKC Inc.) The trap was inserted into a short path thermal desorption injector (TD-2, Scientific Instrument Services, Inc.) and desorbed for 5 min at 200°C using a flow of helium (2 mL/min) into the GC-MS (Agilent 6890GC/5973 MS) injection port that was also set at 200°C. The desorbed VOCs were collected on the column during the desorption process by cooling a small section of the capillary column with an ethanol/dry ice bath (−20°C to −40°C). The column was then equilibrated to 35°C and the temperature program on the GC-MS was started. The compounds were separated using a 30 m × 0.25 mm i.d., 0.25 μm BPX-5 column (SGE), which was set at 35°C for 2 min and increased at 7°C/min until 250°C, and held for 10 min. The mass spectrometer was set to record between 45 and 400 amu.

### Gas chromatography and mass spectrometry

SPME and solvent extracted samples were analysed using GC-MS (Shimadzu GCMS-QP2010, Kyoto, Japan). The separation of emitted components was achieved using a 30 m × 0.25 mm i.d., 0.1 μm Rt-Stabilwax (Restek, Bellefonte, PA) column with ultra-high purity helium as the carrier gas at a constant flow rate of 1 mL/min. Samples were injected in splitless injection mode with the inlet temperature set to 250°C. The initial oven temperature was set at 35°C and held for 5 min then ramped at 7°C/min to 250°C and held for 10 mins. The desorption time for SPME was 5 min, while for solvent injections 1 μL was injected. The ion source was set at 200°C, and the spectrometer was set to record between 45 and 400 amu [21].

### Data analysis

Headspace and supernatant extractions were performed in at least two independent culture-experiments. Data collection and mass spectra generation were performed using the GC-MS Real Time Analysis software and Postrun application (GCMS solution, version 2.40 Shimadzu Corporation) respectively. For compound identification, mass spectra of the target molecule were screened manually with background subtraction against commercial mass spectral libraries (NIST05, NIST05s, Standard Reference Data Program, Gaithersburg, MD). The VOCs profiles were compared in detail for any unique compounds by utilising the data comparison function in the Postrun software. The top 30 peaks with tentative compound assignment (> 95% similarity) and relative intensities from *P. falciparum* cultures and non-infected erythrocytes are reported.

## Results

### Malaria VOCs assay development

#### Design of culture-VOC capture apparatus

Preliminary studies employed T25 flasks coupled with a rubber stopper with two inlets for the culture and capture of headspace atmosphere (Skinner-Adams T, data unpublished). However, the use of plastic containers and rubber introduces organic contaminants which may interfere with the assay. Therefore, custom-designed glass flasks were created for the *in vitro* capture of headspace VOC experiments.

The prototype 1 sampling unit for VOCs analysis is shown in Figure 1A. The flask size allowed the culture of up to a maximum of 18 mL of parasite-cell-medium suspension. To increase parasite mass and VOCs yield, a second design was proposed featuring a shallow container with a large base-area (Figure 1B). The prototype 2 sampling units were custom-made and equipped with two Duran screw thread tube connections (GL14, Vel, Leuven, Belgium) useful for purge and trap, and SPME sampling. A B-24 joint with a fitted glass stopper improved access to the parasite cultures and changing of media. This was particularly important during the optimization phase of the study in which parasite growth was monitored daily by microscopic examination of blood smears. The new design allowed a larger volume set-up (50 mL) of parasite-cell-media suspension. Cultures of high parasitaemia (>20%) were achieved under the conditions specified above with increased air/medium contact surface available for gas-exchange in the new design.

#### Optimization of culture conditions

Headspace capture of VOCs requires an enclosed culture system, where the atmosphere within the culture container must be optimized for parasite growth. The gas mixture of 1% O_2_ and 5% CO_2_ in nitrogen used for routine cultures (i.e. in a 28.4 L Nalgene dessicator) was suboptimal to sustain *P. falciparum* growth in the present custom-designed containers. On macroscopic examination, RBC became much darker after incubation, likely attributable to inadequate oxygenation. A series of down-sized, volume vs duration of gas injection experiments were conducted with an oxygen monitor to determine optimal gas balance. Optimal conditions for normal parasite replication using prototype 2 were achieved with a new gas mixture of 5% O_2_, and 5% CO_2_ in nitrogen [22].

Routine *in vitro* culture of *P. falciparum* usually maintains a maximum of 5% parasitaemia at 5% haematocrit. Prototype 2 container was more suitable for malaria culture than its predecessor. The initial period for VOCs capture was over 48 hr (i.e. one parasite life cycle), however, the system must remain closed during this time without the required media changes. This approach produced stressed parasites as indicated by the presence of gametocytes by microscopy. Therefore, subsequent capture experiments were set up over the later 24 hr of the developmental cycle where there is maximal parasite metabolic activity. Synchronized *P. falciparum* were cultured at high parasitaemia and pooled into prototype 2 containers at the trophozoite stage (>20% parasitaemia at 1% haematocrit). To promote liberation of VOCs from the parasite-media matrix to the headspace, sample containers were incubated in a shaking incubator at slow rotation (40 rpm) for 24 hr.

### Analysis of volatile organic compounds

**Figure 2:**
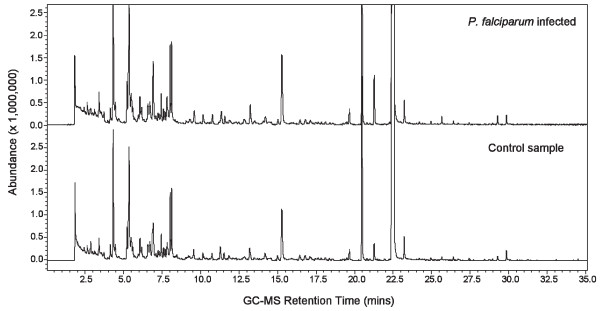
**Chromatograms of VOCs detected in the headspace of cultured *P. falciparum.*** GC-MS total ion chromatogram derived from SPME (triple fibre) sampling of the headspace above *in vitro* cultures of *P. falciparum* infected red blood cells (top) and control red blood cells (bottom). Details of tentatively assigned compounds are presented in Table 1.

This part of the present study employed a number of extraction approaches to capture and analyse VOCs from the culture samples. Headspace VOCs were extracted by SPME (PDMS and combined Carboxen/DVB/PDMS phases – termed triple fibre) and purge and trap coupled with thermal desorption. Direct immersion of the SPME fibre, and traditional extractions with organic solvents with various polarities were used to extract VOCs trapped in the culture supernatant and cell lysate matrix. Overall, mass spectra of over 100 different compounds were detected in the headspace, supernatant and cell lysates of both non-parasitized control and *P. falciparum* cultures. A number of hydrocarbons such as alkanes and alkenes, alcohols, benzene-derivatives and mono-terpenes were identified in both parasitized and control samples (see Figure 2). Table 1 presents the top 30 compounds detected in the samples and relative quantities using the combined triple fibre SPME, which appeared to give the most sensitive results in our experiments. Although minor differences in compound quantities were detected, no unique biomarker for *P. falciparum* was identified, despite detailed comparison of the data files. Similarities between VOCs liberated from non-parasitized control and infected samples were seen using other extraction methods.

**Table 1:** **Representative VOCs detected in control and *P. falciparum *infected cultures using SPME (triple fibre) coupled with GC-MS (Figure**2**)**

		**Relative abundance (%)**	
**Retention time (mins)**	**Compound**	**Control cultures**	***P. falciparum-*infected cultures**
1.81	carbon dioxide	3539462 (1.39)	1554307 (0.62)
2.64	octane	646353 (0.25)	783376 (0.31)
3.40	2,4-dimethyl-1-heptene	623570 (0.24)	615473 (0.24)
4.14	octamethyl tetrasiloxane*	724464 (0.28)	560807 (0.22)
4.33	2,2,4,6,6-pentamethyl heptane	7031090 (2.76)	8250249 (3.28)
4.45	4-methyl nonane	609038 (0.24)	638770 (0.25)
5.22	2,6-dimethyl nonane	1740986 (0.68)	1668518 (0.66 )
5.36	decane	6616172 (2.59)	7986280 (3.18)
5.50	branched alkane (C_11_H_24_)	1452353 (0.57)	1453227 (0.58)
6.07	2,6-dimethyl decane	947057 (0.37)	1061297 (0.42)
6.17	3,7-dimethyl decane	716160 (0.28)	716355 (0.28)
6.59	toluene	1000764 (0.39)	962309 (0.38)
6.70	branched alkene (C_11_H_22_)	1145337 (0.45)	1218163 (0.48)
6.92	unidentified siloxane*	2167416 (0.85)	3703832 (1.47)
7.44	dodecane	983881 (0.39)	1189196 (0.47)
7.82	branched alkane (C_12_H_26_)	698815 (0.27)	1644428 (0.65)
8.03	branched alkene (C_12_H_24_)	2883283 (1.13)	2925879 (1.16)
8.13	branched alkene (C_12_H_24_)	2891039 (1.13)	2873277 (1.14)
9.59	alpha-pinene	516866 (0.20)	762074 (0.30)
10.17	3-heptanone	514761 (0.20)	518528 (0.21)
10.77	isopropyl benzene	404573 (0.16)	498258 (0.20)
11.36	unidentified alkane	910196 (0.36)	725047 (0.29)
11.57	D-limonene	451954 (0.18)	398652 (0.16)
13.22	2-pentyl furan	979481 (0.38)	1209153 (0.48)
15.28	cyclohexanone	3714649 (1.46)	4124560 (1.64)
19.66	cyclohexanol	558229 (0.22)	682196 (0.27)
20.49	1,3-di-tert-butylbenzene	10159046 (3.98)	7908796 (3.15)
21.28	1-octen-3-ol	796995 (0.31)	1909838 (0.76)
22.56	2-ethyl-1-hexanol*	198435706 (77.77)	191847968 (76.31)
23.23	unidentified alkane	1285623 (0.50)	1027694 (0.41)

*known contaminants.

Compounds were tentatively identified (> 95% similarity) by comparing the experimental mass spectra obtained with known compounds present in a commercial library of mass spectra (NIST05).

## Discussion

Despite a number of attempts following a step-wise approach, the present *in vitro* study revealed no specific patterns of VOCs released by *P. falciparum* cultures. Various methods of headspace analysis as well as solvent extraction (hexane, dichloromethane, ethylacetate) of supernatants and cell lysates were examined, but the results showed no obvious differences between *P. falciparum* and control non-parasitized cultures. When used with a pre-concentration device such as SPME, GC-MS has sufficient sensitivity in the low ppt range [23,24] to detect such differences. Despite this sensitivity, data from SPME using PDMS and triple fibre (PDMS + divinylbenzene + carbowax) revealed the production of a variety of VOCs that were derived from background red blood cell cultures. Thermal desorption of purge and trap samples also showed no significant differences with a similar VOCs profile to that observed using SPME fibres, suggesting that both techniques were detecting the majority of released VOCs. Thus, no unique compounds derived from *P. falciparum* were able to be identified above control levels despite a number of methods being investigated.

Previous studies of VOCs generated by other infectious agents have shown positive associations between VOCs liberated *in vitro* and those detected in the breath from patients. This *in vitro* vs *in vivo* relationship is exemplified by studies of respiratory infections with *Aspergillus fumigatus *[15,24,25] and pulmonary tuberculosis [10]. There were, however, a number of important differences between these studies and the present series of experiments. Firstly, bacterial and fungal colonies are cultured on solid medium. Any VOCs produced by bacteria and fungi are released directly into the culture headspace. By contrast, *P. falciparum* are enveloped by two extra layers, being within RBC that settle at the bottom of liquid medium in the culture plate. The possibility of loss of VOCs of malarial origin in cell membranes and the culture medium prompted repeated extractions of supernatant and cell lysate using various organic solvents. No obvious differences between malaria and control cultures were found in these experiments.

Secondly, the biomass of bacteria and fungi *in vitro* assessed from colony-forming units (cfu), viable bacterial or fungal cells per visible colony is much greater than that of *P. falciparum* in a typical culture. The number of cfu per visible colony is substantially higher than the number of malaria parasites in a comparable volume of RBC. For *Mycobacterium tuberculosis* culture, an inoculum of 0.5 mL of a 1.0 McFarland standard contains 1.5 x 10^8^ cfu) [10] compared to a 50 mL *P. falciparum* culture of 20% parasitaemia at 1% haematocrit which contains approximately 1.1 x 10^7^ parasitized cells. In addition, the greater biomass for bacteria housed within a smaller culture vessel (1 mL headspace) considerably increases the concentration and thus likelihood of VOCs detection compared with the larger headspace (500 mL) of an intra-erythrocytic parasite culture.

The analysis of VOCs released from *in vitro* malaria cultures presented a substantial challenge because of the fastidious nature of *P. falciparum* in culture, including microaerophilic requirements, daily medium changes and the need for a closed-system for headspace capture. Although the use of glass culture-capture apparatus minimized the presence of contaminants related to the use of plastics, a number of external VOCs were present. Compounds such as 2-ethyl-1-hexanol (Table 1) may have been derived from bis-2-ethyl-hexyl-phthalate, a common additive to plastics which renders the plastic more flexible [26]. Siloxane derivatives were also present and were derived either from the PDMS coating of the SPME fibre, the GC column stationary phase or silicon septa used to seal the GC injection port.

A number of different gas mixtures have been used to support malaria culture. Atmospheres with combinations of 0.5 to 21% O_2_ mixed with 1 to 7% CO_2_ diluted in nitrogen and microbial gas sachets have been employed [20,27-30]. High oxygen concentrations are considered to cause deleterious effects on parasites and reduce yields [31], however this has been debated [20]. A mixture of 5% O_2_ with 5% CO_2_ in nitrogen supports malaria growth better than 5% CO_2_ with 95% air [32], although both mixtures have been successful [20,28,30,33,34]. Therefore, although gas composition is an important consideration, it should not be singled out as the determining factor for successful cultures, particularly for high parasitaemias. In addition to the use of premixed gas, the transition from 5% haematocrit for routine *P. falciparum* culture to a lower haematocrit (1%) proved an important modification to support high parasitaemia for the VOCs experiments. An appropriate ratio of medium to cell pellet volume prevents parasite toxicity and helps maintain viability [20]*.*

Although VOCs may be more readily detected *in vivo* in respiratory diseases [7,23,25,35], VOCs generated as part of host response to a systemic disease may also serve as biomarkers. Examples include increased production of pentane and carbon disulfide in the breath of patients with schizophrenia [36]. Nevertheless, their specificity remains questionable, as breath carbon disulfide has been detected in both smokers and non-smokers [37] and has been linked with myocardial infarction [9]. An assessment of a characteristic VOCs fingerprint in the context of malaria (including severe and non-severe cases) *in vivo* was beyond the scope of the present *in vitro* experiments reported in this study.

## Conclusions

The present study used optimized experimental conditions to enable the capture, extraction and analysis of VOCs liberated from *P. falciparum* cultures. Even at high parasitaemia (~20%), VOCs unique to *P. falciparum* cultures were not detected using solvent extraction, purge and trap-thermal desorption, or SPME. GC-MS data revealed a variety of VOCs but no unique malarial finger-prints. Future *in vivo* studies analysing the breath of patients with severe malaria may yet reveal specific clinically-useful volatile biomarkers. A child weighing 15 kg with a circulating blood volume of 1 litre sustaining a low 0.2% parasitaemia will, for example, harbour up to 1 x 10^10^ parasites vs only 1.1 x 10^7^ parasites in our *in vitro* system. Notwithstanding the complex kinetics governing VOCs in expired air from such a patient, it is possible that the greater *in vivo* biomass may improve the sensitivity of subsequent detection of VOCs from breath samples relative to that achieved in our *in vitro* experiments. In addition, specific VOCs may be generated *in vivo* in response to the infection.

## Abbreviations

GC-MS: Gas chromatography–mass spectrometry; PDMS: Polydimethylsiloxane; RBC: Red blood cells; SPME: Solid phase micro-extraction; VOCs: Volatile organic compounds.

## Competing interests

The author(s) declare that they have no competing interests.

## Authors’ contributions

RW participated in study design, experimentation, data analysis and drafted the manuscript. GF participated in study design, GC-MS optimization, data analysis and critical revision of the manuscript. TD conceived the study, and participated in its design and coordination, and critical revision of the manuscript. All authors read and approved the final manuscript.
